# Effectiveness of Erector Spinae Muscle Block in Vertebral Oncologic Fracture

**DOI:** 10.7759/cureus.55599

**Published:** 2024-03-05

**Authors:** Lizeth Castillo Ramirez, María del Rocío Guillen Nuñez, Àngel Manuel Jùarez Lemus, Ricardo Plancarte Sànchez, Viviana Andrea Villar Herrera, Gian Marco Gutierrez Herrera

**Affiliations:** 1 Medicina del Dolor, Instituto Nacional de Cancerología, Ciudad de México, MEX; 2 Pain Medicine, Clinica Alive, Ciudad de México, MEX; 3 Medicina del Dolor, Instituto Nacional de Cancerología, Cuidad de Mèxico, MEX; 4 Medicina del Dolor, Instituto Nacional de Cancerología, Ciudad de Mèxico, MEX

**Keywords:** visual analog scale, oral opioid morphine milligram equivalent day, oncology, vertebral fracture, erector spinae muscle block

## Abstract

Objectives

An observational, retrospective, longitudinal, and analytical study aimed to evaluate the effectiveness of the erector spinae plane (ESP) block in managing pain in patients with vertebral fractures secondary to tumoral activity. This study included patients treated at the Pain Clinic who underwent ESP block. The objectives were to describe demographic characteristics, oncological diagnosis, vertebral fracture features, imaging techniques, medications used, and the level of ESP block. Additionally, pain levels were assessed using a numerical analog scale, and the consumption of opioid analgesic medications before and after the ESP block, during follow-up consultations, along with patient satisfaction.

Methodology

This retrospective, observational, and analytical study was conducted at the Pain Clinic of the National Cancer Institute of Mexico. Patients with vertebral fractures secondary to tumor activity were included, with data collected from March 2020 to September 2023. A consecutive non-probabilistic sampling method was employed, and specific inclusion and exclusion criteria were applied. Data were analyzed using descriptive statistics and the Wilcoxon signed-rank test for quantitative variables, with a significance level of p ≤ 0.05. IBM SPSS Statistics v. 26.0 (IBM Corp., Armonk, NY) software was utilized.

Results

A sample comprising 16 individuals was obtained, with an equal distribution between males and females. Fracture levels displayed variation, with L3 (12.5%) and T6 (12.5%) being the most prevalent. The ESP approach was primarily conducted using ultrasound (68.8%), while fluoroscopy and computed tomography were utilized in 25.0% and 6.3% of cases, respectively. Predominantly, methylprednisolone and ropivacaine (75.0%) were administered, with phenol used in 18.8% and a combination of methylprednisolone and bupivacaine in 6.3%. Patient satisfaction levels were reported at 81.3% (satisfied or very satisfied). Statistically significant disparities were noted between baseline and incidental pain reduction and oral opioid equivalent dosage in milligrams of morphine per day (MME/day) before and after ESP block (p ≤ 0.05).

Conclusions

This research provides promising preliminary evidence supporting the effectiveness of ESP block for pain management in vertebral fractures secondary to tumoral activity, enhancing the quality and safety of care for oncology patients. The absence of complications, significant improvement in pain, and reduction in opioid dependence underscore the clinical relevance of this therapeutic approach.

An observational, retrospective, longitudinal, and analytical study aimed to evaluate the effectiveness of the ESP block in managing pain in patients with vertebral fractures secondary to tumoral activity. This study included patients treated at the Pain Clinic who underwent ESP block. The objectives were to describe demographic characteristics, oncological diagnosis, vertebral fracture features, imaging techniques, medications used, and the level of ESP block. Additionally, pain levels were assessed using a numerical analogue scale, and the consumption of opioid analgesic medications before and after the ESP block, during follow-up consultations, along with patient satisfaction.

## Introduction

Cancer has become a major global challenge in public health, with an estimated 190,000 new cases and over 83,000 deaths from cancer each year in Mexico, making it the third leading cause of mortality in the country. The skeletal system is the third most commonly affected organ by metastatic cancer. These metastases are 40 times more common in primary bone tumors of the skeleton, and 65% of them are located in the spine. The thoraco-lumbar region accounts for 70% of these cases, the lumbosacral segment 22%, and the cervical spine 8% [1-3].

Up to 70% of patients who die from cancer have been found to have spinal metastases in autopsies, and 15% present clinically symptomatic disease before dying. The most common cancer diagnoses that lead to spinal metastases are breast, prostate, colon, lung, kidney, and hematopoietic tumors [4].

Pain is the most common symptom in cancer patients, present in 70% to 90% of those in advanced or terminal stages of the disease. In 80% of cases, the pain is directly caused by the tumor, while in 20% it is a consequence of antineoplastic treatment. Pain associated with vertebral fractures occurs through three different mechanisms: intraosseous tumor growth, extraosseous growth toward neighboring paravertebral tissues, and intracanal growth (causing spinal cord or nerve root compression). The pain is generated by increased intraosseous pressure, periosteal expansion, and invasion of neighboring tissues. It also arises from fractures in a pathological area and from vertebral instability secondary to the fracture, causing compression of the spinal cord or nerve root [5].

The initial pharmacological treatment of vertebral fractures consists of the use of NSAIDs, opioids, corticosteroids, and radiotherapy. If associated with neurological compression, it is difficult to control even with optimal multimodal pain management plans, and those patients rarely qualify for other interventional procedures such as osteocementoplasty, which leads to issues in patients' quality of life [6,7].

Vertebral fractures can be treated using vertebroplasty and kyphoplasty techniques; however, not all patients are candidates for these procedures. A recent Cochrane review does not support the routine use of vertebral augmentation techniques (VAT) in patients with vertebral fractures, especially in cases where the pain arises from the posterior elements (lamina and facet joints), particularly the joints above the fracture for anterior wedge compression, and the joints below the fracture for vertical compression, and potentially both joints above and below the fracture [7].

Erector spinae plane (ESP) was initially described as an analgesic technique for the treatment of neuropathic pain but has later been used in specific surgical procedures (mastectomy, thoracotomy, nephrectomy, etc.) with good results. It was first described by Forero et al. in two patients with severe chronic thoracic neuropathic pain and two patients undergoing thoracoscopic surgery. This block is achieved by the action of the local anesthetic on the dorsal and ventral branches of thoracic spinal roots at different levels (through craniocaudal diffusion) and posterior diffusion to the paravertebral space [8-10].

Currently, there are only case reports on the use of ESP for the treatment of pain caused by non-oncological vertebral fractures, but it has a wide range of applications for acute and chronic pain control, making it a viable alternative for pain management in patients with oncological vertebral fractures who are not candidates for other interventional treatments.

## Materials and methods

Study design

An observational, retrospective, longitudinal, and analytical study was carried out at the Pain Clinic of the National Cancer Institute, Mexico. The study included patients with vertebral fractures secondary to tumor activity who met the inclusion criteria. The data were collected from March 2020 to September 2023, with approval by the Research Committee with registration number No. Ref/INCAN/CI/0880/2023.

A consecutive non-probabilistic, intentional, or convenience sampling was employed. The study aimed to demonstrate a difference of at least two points in the pain level between pre- and post-treatment in patients with vertebral fracture secondary to tumor activity, who underwent an (ESP) block approach. A minimum of 20 patients was required, considering a 20% loss.

Inclusion criteria

Patients with a confirmed diagnosis of tumor-related vertebral fracture, radiological evidence of tumor-related vertebral fracture, age 18 years and above, treatment at the Cancer Pain Clinic within the specified period, inadequate pain control with conventional pharmacological treatments, and complete medical records were included.

Exclusion criteria

Patients under 18 years of age, inability to follow up clinically during the study period, fractures unrelated to tumor activity, incapacity to communicate or register pain levels accurately, and severe comorbidities were excluded.

Methodological description

Subjects were selected through consecutive non-probabilistic sampling. Data were collected from the Cancer Pain Clinic's records, and patients meeting inclusion and exclusion criteria were included. Patient data, including sociodemographic information and study variables, were recorded in specially designed data collection sheets, coded, and entered into a spreadsheet for analysis. Statistical analysis was conducted using IBM SPSS Statistics v. 26.0 (IBM Corp., Armonk, NY).

Statistical analysis plan

Descriptive statistics were used to analyze independent and dependent variables. The Wilcoxon signed-rank test was employed for inferential analysis of quantitative variables, with significance set at p ≤ 0.05. Data were processed with a 95% confidence interval and α level of 0.05.

This study's design and methodology provide a comprehensive framework for evaluating the effectiveness of treatment in cancer-related vertebral fractures. The statistical analysis plan ensures robustness in drawing conclusions from the data collected.

## Results

Demographic characteristics

**Figure 1:**
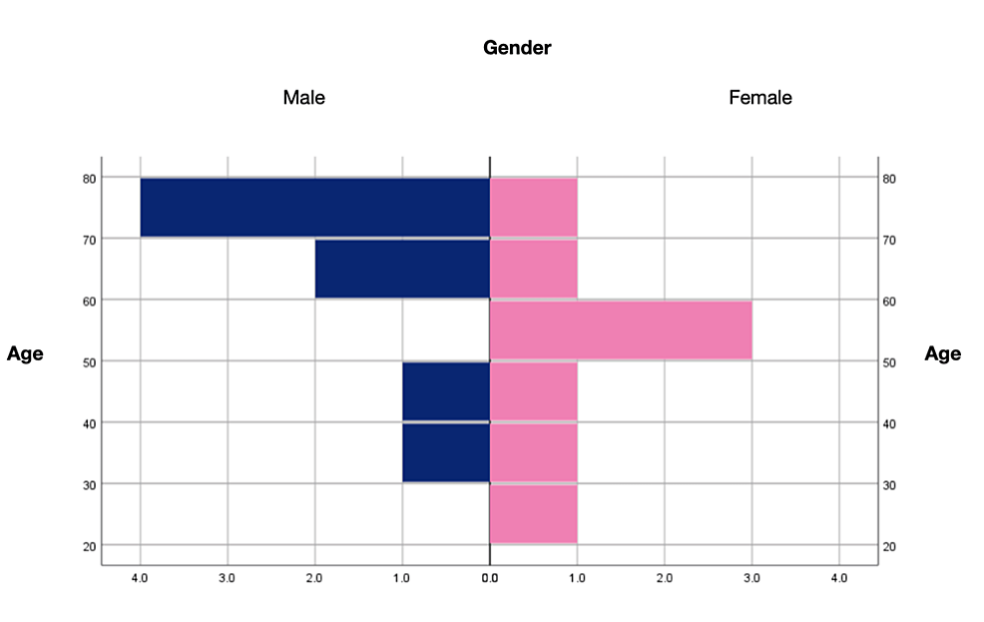
Histogram of gender and age in the sample

A total of 16 patients were included, evenly divided between women (eight, 50%) and men (eight, 50%). The age ranged from 21 to 75 years with an average of 56.8 years (Figure 1).

The most frequent oncological diagnosis was breast cancer (four, 25.0%), followed by prostate cancer (three, 18.8%), and multiple myeloma (two, 12.5%) (Table 1).

**Table 1:** Oncological diagnosis

Variable		Counting	Percentage
Oncological diagnosis	Breast Cancer	4	25.0
	Prostate Cancer	3	18.8
	Multiple myeloma	2	12.5
	Malignant Lip Cancer	1	6.3
	Malignant Retroperitoneum Tumor	1	6.3
	Thyroid Cancer	1	6.3
	Mesothelioma	1	6.3
	Gastric cancer	1	6.3
	Rectal Cancer	1	6.3
	Breast cancer + basal cell	1	6.3
	Total	16	100.0

ESP block characteristics

The approach to the ESP block was predominantly ultrasound-guided (11, 68.8%), fluoroscopy was used in four patients (25.0%), and computed tomography in one (6.3%). The most frequently targeted level for the block was bilateral L3 (three, 18.8%), bilateral L2 (two, 12.5%), and bilateral T7 (two, 12.5%). Cases were also observed at bilateral L4, left L4 - right L5, left T1, bilateral T4, left T5, left T6, left T7 - right T6, bilateral T9, and right T9 levels (one, 6.3% each). The transverse process of the targeted vertebra was selected, and after asepsis and antisepsis, the three layers of the ESP muscles were identified. A Whitacre 22-gauge needle was inserted, and the medications used were methylprednisolone and ropivacaine in most patients (12, 75.0%), phenol in three patients (18.8%), and methylprednisolone and bupivacaine in one patient (6.3%). The dose of ESP block ranged from 10 to 30 mL with an average of 16.4 mL. No complications were reported during or after the procedure (0%) (Table 2).

**Table 2:** ESP block characteristics

Variable		Counting	Percentage
Lock Level	L3 Bilateral	3	18.8
	T7 Bilateral	2	12.5
	L2 Bilateral	2	12.5
	T9 Right	1	6.3
	T9 Bilateral	1	6.3
	Left T7 and Right T6	1	6.3
	Left T6	1	6.3
	Left T5	1	6.3
	T4 Bilateral	1	6.3
	Left T1	1	6.3
	Left L4 - L5	1	6.3
	L4 Bilateral	1	6.3
	Total	16	100.0
ESP technique	Ultrasound	11	68.8
	Fluoroscopy	4	25.0
	Tomography	1	6.3
	Total	16	100.0
Medication used	Methylpredisolone 20-40 mg + Ropivacaine 0.2 %	12	75.0
	Phenol 6-8 %	3	18.8
	Methylpredisolone 20 mg + Bupivacaine 0.25 %	1	6.3

Pain assessment before and after ESP block

**Figure 2:**
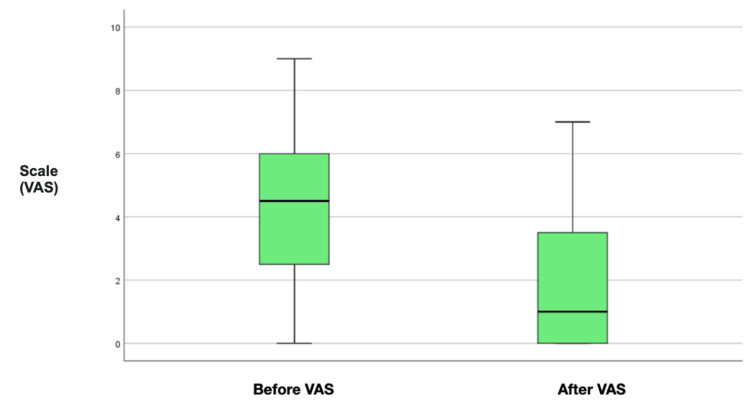
Differences between pain VAS before and after ESP block.

Considering the Visual Analog Scale (VAS), before the ESP Block, the average baseline pain level was 4.3, and the average incidental pain was 7.8. Compared to the pain level after the ESP Block, there was a reduction in the average baseline pain by 1.9 and in incidental pain with an average of 4.8 (Figure 2).

Assessment of MME/day before and after ESP block

**Figure 3:**
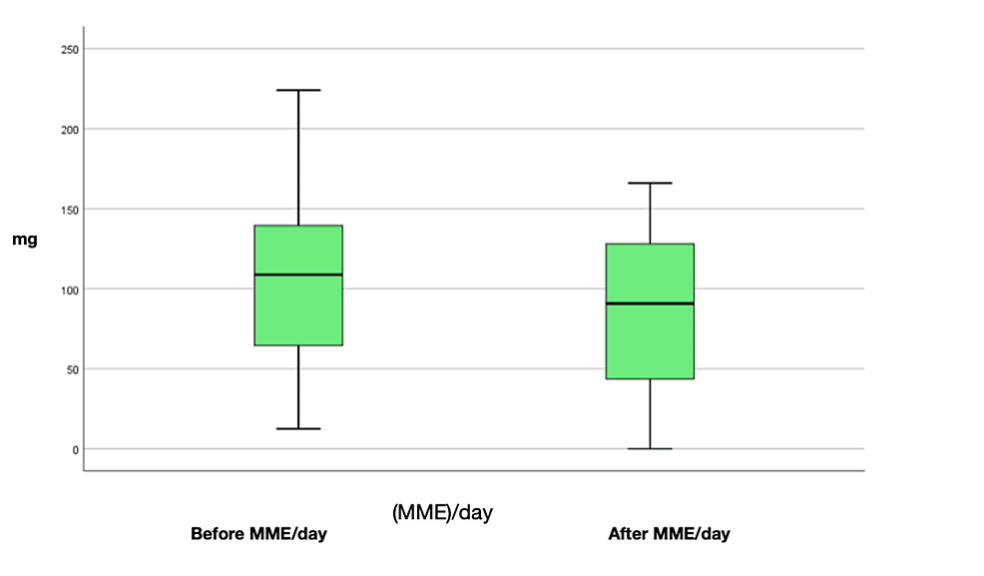
Differences between MME/day before and after ESP block.

The average MME/day before the procedure was 105.2 mg, with a reduction in MME/day averaging 85.8 mg (Figure 3).

Satisfaction level assessment

When patients were asked to self-assess their pain seven days after the block using a Likert scale, the majority reported being completely satisfied (seven, 43.8%), satisfied (six, 37.5%), neutral (two, 12.5%), and dissatisfied (one, 6.3%) (Table 3).

**Table 3:** Satisfaction level assessment

Variable		Counting	Percentage
Satisfaction	Totally satisfied	7	43.8
	Satisfied	6	37.5
	Nether satisfied nor dissatisfied	2	12.5
	Dissatisfied	1	6.3
	Totally dissatisfied	0	0.0
	Total	16	100.0

Differences between pain and MME/day before and after ESP block

Statistically significant differences were found between baseline pain before and after the procedure (Z = -3.13, p = 0.001) and between incidental pain before and after the procedure (Z = -3.32, p = 0.001), correlated with MME/day before and after the procedure (Z = -2.7, p = 0.007). The use of the Wilcoxon signed-rank test for related samples demonstrates statistically significant differences, underscoring the robustness of the findings of this study. The results suggest that the ESP is a safe and effective intervention for pain relief in patients with vertebral fractures. These findings support its integration as part of a multimodal approach to pain management in this patient population, with the potential to mitigate opioid dependence and offer an alternative or complementary method for pain relief (Table 4).

**Table 4:** Differences between pain and MME/day before and after ESP block

	Mean	Median	Standard Deviation	Minimum	Maximum	IQR	P-value
Before VAS (average)	5.3	6.0	2.1	1.0	8.7	2.7	0.001*
After VAS (average)	2.7	2.3	1.9	0.0	6.0	3.1	
Before MME/day (mg)	105.2	108.8	59.6	12.5	224.0	85.5	0.007*
After (MME/day) (mg)	85.8	90.8	49.1	0.0	166.0	86.3	

## Discussion

The ESP block has emerged as a promising technique in the pain management arsenal, especially in the context of spinal procedures and trauma. The provided information details the anatomical foundations, application technique, and potential benefits of the ESP block [9].

One highlighted aspect is the versatility of the ESP block in terms of compartmental extension and application at different vertebral levels. The technique has proven to be effective in both the lumbar and thoracic spine, demonstrating its ability to provide analgesia over a wide area, including specific dermatomes. The safety of the technique, especially in anticoagulated patients, is a significant advantage. The lack of noticeable motor or sensory block, coupled with a lower risk of neurovascular and pleural injuries, contributes to the prominent position of the ESP block compared to other neuraxial options, such as epidural or paravertebral blocks [10-16].

However, despite the advances and evident benefits, it is essential to address the limitations and areas of uncertainty associated with the ESP block. Variability in block quality between lumbar and thoracic regions highlights the need for a deeper understanding of anatomy and local anesthetic spread in these different spinal segments [17,18].

Regarding the management of difficult-to-control chronic pain, there are few case reports associated with ESP block combined with the use of neurolytics. One such report was published as a letter to the editor, conducted at the National Cancer Institute by Hernandez et al., achieving significant pain relief for a short duration. This served as a turning point to continue with study protocols for neurolytic block use, requiring randomized clinical trials to assess efficiency and safety as an alternative in intractable cancer pain [19].

As the ESP block continues to gain acceptance in acute perioperative pain management, it is imperative to direct future research toward standardizing the technique, determining optimal doses, and evaluating its effectiveness in specific conditions, such as chronic pain associated with vertebral fractures in oncology patients. This evidence-based approach is essential to solidify the ESP block's position as a valuable tool in pain control [20-23].

In conclusion, while retrospective studies provide valuable information, moving towards prospective research is essential to enhance our understanding of the efficacy of ESP block and refine its clinical application. By addressing methodological limitations and conducting well-designed prospective studies, we can further optimize the ESP block technique, leading to improved outcomes, providing alternative options in interventional pain management, and thereby enhancing patient care.

## Conclusions

Treatment of chronic pain in cancer patients, particularly when the disease progresses to metastasize to other organs, poses a significant challenge due to the increased use of analgesics and associated side effects. The ESP block has become an integral part of the National Cancer Institute's interventional treatment for patients with oncologic bone dissemination in the spine and accompanying vertebral fractures.

Despite the obvious benefits and advances associated with ESP block, it is essential to recognize its limitations and areas of uncertainty, the variability in the quality of the block between the need for a deeper understanding of the anatomy and the distribution of the local anesthetic in the different segments of the spine.

Addressing these limitations through further research aimed at standardizing the technique, optimizing doses, and evaluating its effectiveness in specific conditions, such as chronic pain associated with vertebral fractures in oncology patients, is crucial to solidifying the position of ESP block as a valuable tool in pain management.
